# Statistics of Medical Colleges

**Published:** 1846-07

**Authors:** 


*Statistics of Medical Colleges.*—We have deferred publication of the number of pupils and graduates of medical schools for the past season, in order to collect reports from all the institutions of our country, and present them collectively. We give below a list of those of which we have been able to obtain information.

| | *Class* 1845–6. | *Graduates.* |
|---|---|---|
| University of Pennsylvania, | 471 | 168 |
| Jefferson Med. College, Philadelphia, | 459 | 170 |
| University of City of New-York, | 425 | 131 |
| College Physicians and Surgeons, N. Y., | 200 | 38 |
| Geneva Med. College, N. Y., | 178 | 39 |
| Albany Med. College, N. Y., | 115 | 42 |
| Harvard University, Boston, Mass., | 159 | 31 |
| Berkshire Med. Institution, Mass., | 142 | 35 |
| Castleton Med. College, | 140 | 36 |
| Yale Med. College, New Haven, Conn., | 53 | 19 |
| Cleveland Med. College, Ohio, | 160 | 52 |
| Willoughby Med. College, Ohio, | 164 | 40 |
| Vermont Med. College, Woodstock, | | 24 |
| Ohio Med. College, Cincinnati, | 195 | 46 |
| Transylvania Med. College, Lexington, Ky., | 171 | 64 |
| Louisville Med. Institute, Ky., | 345 | 43 |
| University of Maryland, Baltimore, | 147 | 40 |
| Bowdoin Med. College, Brunswick, Me., | 73 | 19 |
| Rush Med. College, Chicago, Ill., | 50 | 9 |
| Indiana Med. College, La Porte, | 81 | 18 |
| Medical College of Louisiana, New Orleans, | 103 | 20 |
| Medical College of Georgia, Augusta, | 112 | 15 |
| Missouri University, St. Louis, | | 29 |
| Kemper College, St. Louis, | | 11 |
| Total number of pupils, | 3,954 | |
| Total number of graduates. | | 1,359 |

It will be perceived that in a few instances the reports are incomplete. There are several institutions, also, not included in the list from our not receiving catalogues, and not being able to derive information from any available sources.

